# Vaccine Technologies and Platforms for Infectious Diseases: Current Progress, Challenges, and Opportunities

**DOI:** 10.3390/vaccines9121490

**Published:** 2021-12-16

**Authors:** Majed Ghattas, Garima Dwivedi, Marc Lavertu, Mohamad-Gabriel Alameh

**Affiliations:** 1 Department of Chemical Engineering, Polytechnique Montreal, Montreal, QC H3T 1J4, Canada; majed.ghattas@polymtl.ca; 2 Institute of Biomedical Engineering, Polytechnique Montreal, Montreal, QC H3T 1J4, Canada; 3 Department of Biological Engineering, Massachusetts Institute of Technology, Cambridge, MA 02139, USA; gdwivedi@mit.edu; 4 Division of Infectious Diseases, Perelman School of Medicine, University of Pennsylvania, Philadelphia, PA 19104, USA; 5 AexeRNA Therapeutics, Washington, DC 20001, USA

**Keywords:** vaccine, vaccine types, vaccine platforms, inactivated vaccine, live attenuated vaccine, Virus-like Particles, toxoid vaccine, polysaccharide vaccine, next generation vaccines, viral vectored vaccine, DNA vaccine, mRNA vaccine

## Abstract

Vaccination is a key component of public health policy with demonstrated cost-effective benefits in protecting both human and animal populations. Vaccines can be manufactured under multiple forms including, inactivated (killed), toxoid, live attenuated, Virus-like Particles, synthetic peptide, polysaccharide, polysaccharide conjugate (glycoconjugate), viral vectored (vector-based), nucleic acids (DNA and mRNA) and bacterial vector/synthetic antigen presenting cells. Several processes are used in the manufacturing of vaccines and recent developments in medical/biomedical engineering, biology, immunology, and vaccinology have led to the emergence of innovative nucleic acid vaccines, a novel category added to conventional and subunit vaccines. In this review, we have summarized recent advances in vaccine technologies and platforms focusing on their mechanisms of action, advantages, and possible drawbacks.

## 1. Introduction

Vaccine administration, also known as vaccination, is one of the most effective approaches to prevent and control serious, and sometimes deadly, infectious diseases. Surveillance, ring, and mass vaccination campaigns have helped eradicate diseases such as smallpox [1] and significantly reduce morbidity and mortality caused by several pathogens including SARS-CoV-2 [2]. In the United States and other high-income countries, vaccines have proven instrumental in eliminating debilitating diseases such as poliomyelitis, *haemophilus influenzae b* (Hib), rotaviral enteritis, hepatitis, parotitis (mumps), pertussis, varicella (chickenpox), tetanus, measles, and diphtheria. According to the World Health Organization (WHO), vaccines prevent between 2–3 million deaths every year despite the considerable disparity in access to vaccines, especially in low- and middle-income countries [3].

As evident from the Ebola epidemic, and the COVID-19 pandemic, emerging infectious diseases with high incidence or fatality rates pose a substantial threat to public health in addition to the local and global economy. The ability to respond to such threats in a short period depends on multiple factors including monitoring, pandemic preparedness programs, government/non-government cooperation, national policies, and current technologies and platforms to produce and distribute vaccines. Immediate response strategies such as social distancing, quarantine, and lockdown, control the spread of an emerging pathogen effectively but are practically challenging to enforce long-term. Rapid rollout of mass vaccination programs reduces treatment costs and prevent overwhelming the healthcare system by limiting disease transmission, preventing the clinical manifestation of infection in a short period, and establishing herd immunity.

Vaccine manufacturing technologies and platforms have evolved over the years to overcome limitations, reflect technological advancements, and address ongoing concerns. The goal of this review is to provide an overview of our current understanding of vaccine technologies and platforms with a focus on immune response mechanisms, benefits, and potential limitations of conventional and next-generation vaccine technologies and platforms. We believe that this review will assist in improving understanding of the safety and benefits of vaccination for a broad section of society and potentially reduce misinformation and hesitation associated with vaccination.

## 2. What Are Vaccines?

**Figure 1:**
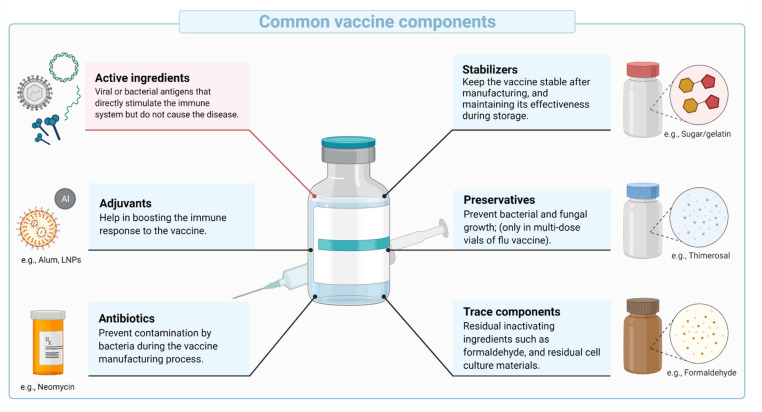
Schematic representation of common vaccine components, showing the typical vaccine components, including the active ingredients, stabilizers, adjuvants, preservatives, antibiotics, and trace components.

Vaccines are biological compositions intended to stimulate and prepare the immune system against infection or disease. They utilize the ability of the highly evolved mammalian immune system to recognize, respond to and remember pathogens. The primary component of vaccines are antigens that are derived from the pathogen of interest or biomanufactured. Additional components may include preservatives, stabilizers, excipients, and traces of products carried over during manufacturing (Figure 1). Often, adjuvants are added to improve immunogenicity (ability to induce an immune response) and efficacy in some populations (e.g., infants, elderly, and immunocompromised individuals) and/or to allow antigen dose sparing (increase the global supply of vaccines).

Vaccines may be applied as a prophylactic or therapeutic modality. Prophylactic vaccines prevent disease through the administration of antigens to healthy individuals and are primarily developed for infectious diseases. Prophylactic vaccines are generally designed to generate antibodies that neutralize the pathogen (neutralizing antibodies or NAbs). In contrast, therapeutic vaccines are administered as a post-exposure therapy to bolster the subject’s immune system against a chronic infection, premalignant condition, or a preexisting disease such as cancer. They are designed to induce cell-mediated immunity resulting in the elimination of infected cells. Therapeutic vaccines are out of the scope of this review paper and the interested reader is referred to an excellent review by Saxena et al. [4].

Unlike therapeutic drugs meant for ailing individuals, vaccines are typically administered to infants and healthy adults. They are subject to stringent testing and quality standards from local and international regulatory agencies such as the United States Food and Drug Administrations (FDA), the European Medicine Agency (EMA), and WHO, to ensure efficacy and safety. Pre-clinical and clinical vaccine data are closely monitored before recommendation for public use followed by post-market surveillance to continuously evaluate the safety, efficacy, and any long-term side effects.

## 3. Immune Responses to Vaccines

**Figure 2:**
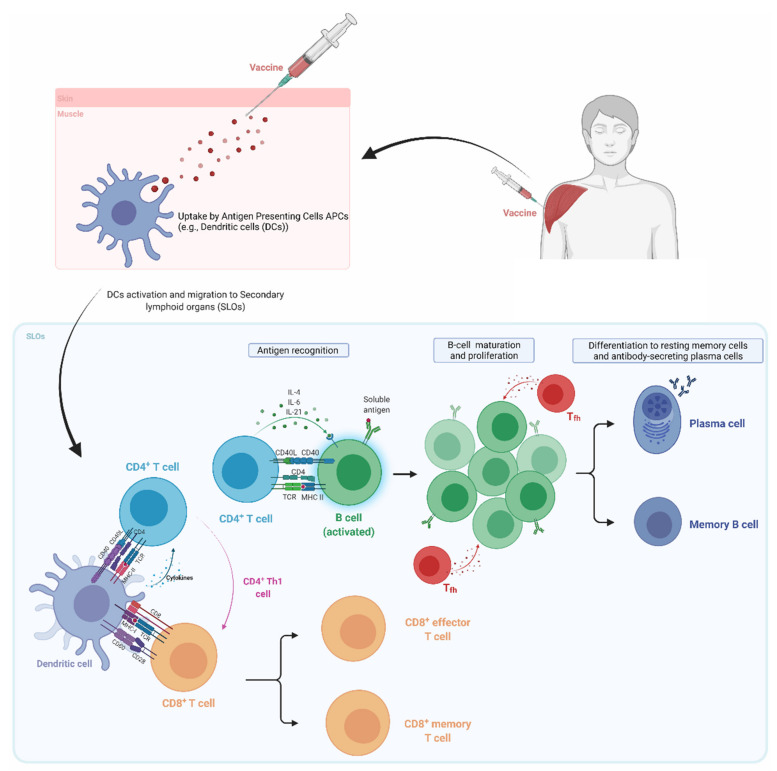
Basics of the immune response to vaccines following intramuscular administration. Vaccine components (e.g., antigen, and/or adjuvant) are recognized and phagocytosed (or uptaken) by tissue resident innate immune cells, or antigen presenting cells (APCs), such as dendritic cells (DCs) and macrophages (Mϕs). The process of antigen and/or adjuvant recognition, phagocytosis, and intracellular processing of antigens induce APCs to mature (e.g., increased expression of clusters of differentiation (CDs) such as CD80, CD40, MHC…), and migrate to secondary lymphoid organs (SLO; e.g., draining lymph nodes (dLN), and the spleen). Incoming APCs encounter and interact with T lymphocytes through molecular recognition between the APCs major histocompatibility complex (MHC) and the T cell receptor (TCR); also known as signal 1. This interaction is stabilized through an additional set of interactions between receptors, or co-receptors, on both cell types (i.e., CD40-CD40L); also known as signal 2. Interaction between MHC-II and the TCR, co-receptors, and APC secreted cytokines (also known as signal 3) induces the activation of helper T cells (T_h_ or CD4^+^ T cells). In some cases, antigens may be cross-presented on class I MHC in addition to the canonical class II MHC presentation. The former interacts with the TCR of CD8^+^ T cells, leading to their differentiation into effector (cytotoxic) T cells and memory CD8^+^ T cells. CD4^+^ T cells differentiate into one of the subclasses (e.g., Th2, Tfh, Th17, Th9…).

The main objective of a vaccine is to generate a long-lasting antigen-specific protective immune memory. A detailed understanding of the immune system, pathogen biology, and the ensuing host-pathogen interactions is important for vaccine development. Here we introduce concepts of immune responses to vaccines and highlight elements that need to be explored further for the development of vaccine platforms to produce more potent, and safer vaccines (Figure 2).

Following administration, vaccine antigens are recognized by a heterogeneous population of immune cells known as antigen-presenting cells (APCs) comprised of dendritic cells (DCs), macrophages (Mϕ), Langerhans cells, and B-lymphocytes (B cells). Antigen recognition is mediated by a set of proteins capable of sensing macromolecular structures associated with pathogens i.e., Pathogen-Associated Molecular Patterns—PAMPs. These PAMP sensors known as Pattern Recognition Receptors (PRRs) are present on the surface (e.g., Toll-like receptors) and in the cytoplasm (e.g., Retinoic inducible gene I) of APCs (Table 1).

The interaction between vaccine antigens and PRRs on APCs triggers intracellular signaling events that promote phagocytosis, maturation, and secretion of cytokines [12]. Internalized antigens are processed (digested) into peptide fragments and displayed on a set of cell surface receptors known as major histocompatibility complex (MHC). Vaccine antigens that are produced in or enter the cytoplasm (e.g., live attenuated viruses) are displayed on MHC-I through a process known as the endogenous antigen-processing pathway. MHC-I-displayed antigens are recognized by the T cell receptor (TCR) of a particular subset of lymphocytes known as CD8^+^ T cells (cytotoxic T cells—T_c_). In contrast, antigens that enter cells via phagocytosis (e.g., inactivated pathogens, recombinant proteins, or antigens that are secreted/shed from infected cells) are displayed on MHC-II by the exogenous antigen processing pathway and are recognized by another subset of lymphocytes known as CD4^+^ T cells (T helper cells—T_h_) [13]. In some cases, extracellular antigens can also be presented (cross presented) on MHC-I through the vacuolar and/or the endosome-to-cytoplasm cross-presentation pathways [14]. For further details, the interested reader is encouraged to consult a review on the subject [14].

Activated APCs displaying vaccine antigens on MHCs migrate to secondary lymphoid organs such as the draining lymph nodes, and spleen to encounter naïve T cells in specific locations known as T cell zones [15]. Interaction between antigen-presenting APCs and T cells through MHC/TCR binding leads to the differentiation and proliferation of naïve T cells into effector cells. For activation and differentiation, both CD4^+^ and CD8^+^ T cells require two additional signals from co-stimulation and cytokines. Co-stimulation is provided by the interaction of ligands and co-receptors on APCs (e.g., CD80) and T cells (e.g., CD28). In contrast, cytokines are either secreted by the APCs or are present in the microenvironment.

In response to MHC-II/TCR binding, ligand-receptor interaction, and environmental cues from cytokines, CD4^+^ T-helper (T_h_) cells differentiate into distinct effector T_h_ lineages or subsets. T_h_ subsets polarize the immune response through the patterns of cytokines they produce. T helper 1 (T_h1_) and 2 (T_h2_) cells are primarily responsible for cellular and humoral immunity, respectively. T_h1_ cells secrete Interferon-gamma (IFN-γ) as their signature cytokine to stimulate the activation and expansion of cytotoxic T cells. CD8^+^ T cells differentiate into cytotoxic (killer) following TCR/MHC-I interaction, and help from T_h1_ cells (e.g., INF-γ). Induction of cytotoxic T cells following vaccination is important as they recognize and eliminate infected cells, protecting against intracellular pathogens (e.g., viruses). In addition to the effector cells that are generated in response to the presentation and recognition of vaccine antigens, both CD4^+^ and CD8^+^ lymphocytes also differentiate into memory cells (e.g., central memory, effector memory, etc.). The memory cells are critical in responding and expanding the clonal pool upon antigen re-stimulation or subsequent encounter with the pathogen.

Several subsets of T cells play an important role in vaccine-mediated humoral immunity. Th_1_ cells are involved in the production of IgG_1_ and IgG_3_ antibodies by B cells [13]. T_h2_ cells secrete IL-4, IL-5, and IL-13 as their signature cytokines to promote the development, maturation, and differentiation of B cells into memory B cells (MBCs) and antibody-secreting plasma cells (PCs). Follicular T helper (T_fh_), and T_h17_, discovered a few decades ago, are two essential T_h_ subtypes for the generation of high-affinity antibodies and mucosal immunity, respectively. T_fh_ regulates B cell affinity maturation (somatic hypermutation), selection of high-affinity germinal center (GC) B cells, and the duration of GC reaction [16,17]. Durable GC reaction favors the differentiation of GC B cells into high-affinity MBCs and Ab-secreting long-lived PCs (LLPCs) [18,19,20]. MBCs are important in vaccine-induced immunity, as they can rapidly expand, and differentiate into antibody-secreting plasma cells upon antigen re-encounter to provide protection. LLPCs move from the draining lymph nodes (dLNs) germinal centers to the bone marrow to produce antibodies over a few months to decades. LLPCs are terminally differentiated, and in contrast to MBCs do not require reactivation or antigen re-encounter. High levels of neutralizing antibodies produced by LLPCs protect against reinfection.

B cells are also able to recognize and respond to vaccine antigen before the engagement of help from T cells (T_h_). Following vaccine administration, B cells recognize and internalize antigens, and upon PRR activation differentiate into short-lived antibody-secreting cells, plasmablasts, that produce the first wave of antibodies. In the absence of help from T cells (described above), B cells fail to class switch into high-affinity antibody IgG secreting cells and continue to secrete IgM [13].

The above elements of the immune response help guide the development of vaccines and vaccine technologies or platforms to befit the intended use. For instance, the development of vaccines against intracellular infectious agents such as viruses must use technology, or a platform that promotes the endogenous antigen processing pathway or cross-presentation to induce potent cytotoxic T cell responses to eliminate intracellular pathogens. In addition, identification of vaccine technologies or platforms and/or adjuvants that strongly promote T_fh_ cell and GC responses, as well as the LLPCs, is essential to developing effective vaccines to target current and emerging infectious diseases.

In addition to preparing the body against potential infections, vaccines may produce transient mild to moderate side effects. These potential side effects typically manifest between 24–48 h after administration and include injection site pain, soreness, muscle ache, and fever. These manifestations are generated because of the inflammatory component of innate immune cells (e.g., APCs) and indicate that the body is responding to the vaccine. The absence of side effects does not suggest that the vaccine was ineffective, but merely that every human responds differently. Of note, serious side effects are extremely rare, and the protection offered by the vaccine against deadly diseases far outweigh the side effects associated with vaccination.

## 4. Vaccine Types

**Figure 3:**
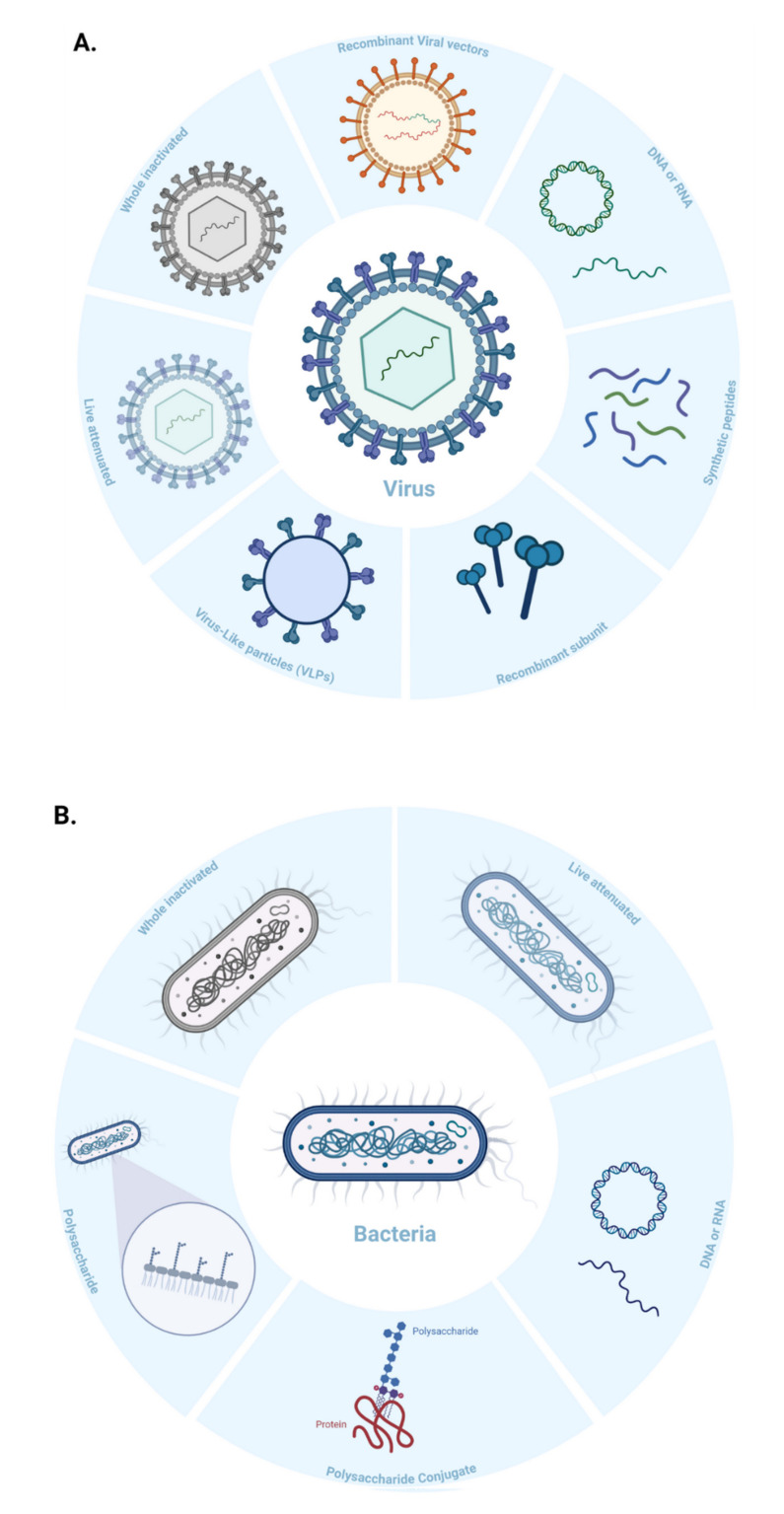
Schematic representation of the different vaccine platforms for infectious diseases, showing different vaccine technologies against (**A**) viral, and (**B**) bacterial pathogens.

Vaccines can be classified based on their ability to replicate in the host (e.g., live *versus* dead vaccines) and/or the technology/platform used in their manufacturing (Figure 3). Conventional vaccines use one or several antigens derived from inactivated or weakened pathogens, or their components such as protein subunits or toxins to generate an immune response. Many licensed vaccines are produced using conventional vaccines technologies. However, these technologies have been unsuccessful in creating vaccines against complex pathogens that can evade the immune system, and/or display extensive variability [21]. Conventional vaccines are also more time-consuming to produce, involve a greater risk of reversion to virulence, and need more customized development against emerging or rapidly evolving pathogens. New strategies for immunogen design and genetic engineering (including recombinant DNA technologies) have contributed to the rise of next-generation vaccine platforms that enable a more potent immune response. Since next-generation vaccines rely on the genetic sequence of a pathogen, they can be developed much faster than conventional technologies. In this manuscript, we have categorized vaccine types based on the manufacturing technology or platform, discuss their production, mechanisms of action, benefits, limitations, and share examples of vaccines licensed for human use.

### 4.1. Conventional (Classical) Vaccine Technologies

#### 4.1.1. Live-Attenuated, or Replication-Competent Attenuated Vaccines

Live attenuated, or replication-competent attenuated vaccines are prepared from weakened pathogens, where the virulence indicated by severity or harmfulness of the disease is considerably reduced. However, the attenuated pathogens mimic natural infection in their ability to infect, replicate, and release in the host [22]. The ability to maintain the pathogens’ replication potential without causing disease or reversion to virulence is a key consideration for this technology.

Attenuation methods involve serial passaging of the virulent pathogens in suboptimal conditions [23,24] or temperatures to induce a selective pressure that favors mutations incapacitating disease potential [22,25,26]. While serial passaging has been applied to develop vaccines for clinical use, other methods such as increasing replication fidelity, and codon de-optimization, are currently being investigated to improve the safety of replication competent attenuated viruses. Using small animal models, Vignuzzi et al. demonstrated that decreasing the number of errors generated from the virus replication machinery (e.g., RNA dependent RNA polymerase) attenuated poliovirus and prevented its reversion to the pathogenic wild-type phenotype [27]. Based on the concept of quasi-species, this method relies on the principle that the pathogenicity of RNA viruses is linked to genome diversity and not necessarily the growth rate. The idea of generalizing the approach of increasing replication fidelity (i.e., reducing error rates) to other viruses is an exciting one but needs further assessment and validation. Another promising method of genetic engineering to attenuate viruses involves altering the positions of synonymous codons to recode the virus genome, thereby increasing the number of suboptimal codon pairs and CpG dinucleotides. This codon de-optimization method was shown to reduce mRNA stability and translation efficiency, in addition to reduced protein production, increased errors in translation, and attenuation of the de-optimized virus [28].

Improved immunogenicity of this vaccine technology is derived from activation of molecular sensors of the innate immune cells (Table 1), sustained antigen expression, and presentation/shedding. Activation of PRRs on DCs induces the upregulation of costimulatory molecules [29], interferon/cytokine expression, and subsequent differentiation and activation of the T_h1_ subset leading to potent cellular immune responses [11]. For instance, the live attenuated yellow fever vaccine 17D (YF-17D) elicits an effective innate immune response through activation of TLR 2, 7, 8, and 9 and the release of pro-inflammatory cytokines such as interleukin IL-12p40, IL-6, and interferon-alpha (INF-α) [30]. Most attenuated vaccines do not need an adjuvant [29] and a single dose is sufficient to confer lifelong immunity. For instance, the smallpox vaccine offers humoral protection for up to 75 years and antiviral T cell protection for up to 15 years [22,31]. The major disadvantage of this technology lies in its disease-causing potential in normal and immunocompromised individuals. Rare cases of disease were recorded following administration of oral poliovirus [22,32,33] and animal rabies live-attenuated vaccines [34,35]. In addition, this technology is labor-intensive, and requires stringent quality control as well as qualified, trained personnel, resulting in increased manufacturing costs and slow response in the event of a pandemic [36]. Despite these disadvantages, live-attenuated vaccines continue to be used since the benefits outweigh the risk of being unvaccinated. Moreover, this technology has led to the successful development of several older and highly effective vaccines protecting from serious diseases (Table 2, e.g., Ebola, Polio, etc.).

#### 4.1.2. Whole Inactivated Vaccines (Killed Vaccine)

Inactivated vaccines are derived from a killed form of virulent pathogens and typically stimulate an antibody-mediated immune response. The inactivation process is mediated by chemical or physical methods or a combination of both. Examples of chemical mediators used for pathogen inactivation include formaldehyde [37], glutaraldehyde [37,38,39], ascorbic acid [40], hydrogen peroxide [41], β-propiolactone [42], and ethylenimine derivatives [43]. Physical inactivation is typically accomplished by heat [44,45] and/or pH denaturation [46,47], ultraviolet light and/or gamma irradiation [48], or other methods [49].

Formaldehyde, or formalin (37% saturated form of formaldehyde), an aldehyde-based cross-linker, has been widely used to inactivate pathogens. Inactivation by formaldehyde involves a multitude of chemical modifications with methylol groups, Schiff bases, and methylene bridges to crosslink biological macromolecules. Formaldehyde inactivation methods differ considerably with respect to formalin concentrations (0.009 to 0.08% ***w/v***), duration of inactivation (days to months), and temperatures (4 or 37 °C). Higher formalin concentrations and temperatures lead to faster inactivation but decrease vaccine efficacy/immunogenicity through increased crosslinking and loss of key epitopes. On the other hand, high temperatures induce accelerated antigen degradation and aggregation [50,51]. As a result, it is important to consider an inactivation period long enough to ensure proper inactivation while maintaining immunogenicity [50].

Whole inactivated vaccines are safer than their attenuated counterparts because inactivation prevents replication and gain of function mutations that could lead to reversion to virulence. These vaccines generate a broad immune response against multiple targets since the whole pathogen is used for immunization. Inactivated vaccines are typically inexpensive to produce and are thermostable, permitting long-term storage. The major drawback of vaccines produced using this technology lies in their limited ability to trigger cellular immune responses against intracellular pathogens. In addition, larger doses and regular booster injections are required for lasting protection due to lower immunogenicity. Higher doses and repeated administration increase potential adverse events and manufacturing costs and reduce vaccine compliance. Notably, the efficacy of inactivated vaccines can be enhanced by increasing the dose or the addition of an adjuvant in the formulation [52]. Finally, chemical, and physical inactivation methods rely on empirical optimization of parameters to achieve a balance between inactivation and immunogenicity. As a consequence of increased development time, research and manufacturing costs are increased which impedes responsiveness to emerging pathogens.

Examples of vaccines prepared by formaldehyde-induced inactivation include Poliovirus (IPOL^®^), Hepatitis A Virus (HAVRIX^®^ and VAQTA^®^), and Japanese Encephalitis Virus (IXIARO^®^) [50] (Table 3). Purified inactivated Zika virus vaccine (PIZV) prepared using 0.02% formaldehyde for 14 days with aluminum hydroxide as an adjuvant is currently being tested in clinical trials. Preclinical immunogenicity and efficacy evaluation in mice showed protection against lethal challenge with the live virus (ZIKV). Additionally, the study showed that unadjuvanted vaccines failed to mount a sufficient humoral response, highlighting the significance of the adjuvants in the formulation of the inactivated vaccines [53]. PIZV vaccine conferred complete protection to rhesus macaque by eliciting a dose-dependent neutralizing antibody response that negatively correlated with the ZIKV RNA after challenge and lasted for at least one year post-vaccination [54]. These studies helped advance this vaccine candidate towards phase 1 clinical trials (NCT03343626), where it was shown to be well-tolerated with an acceptable safety profile. This technology is also being explored for vaccines against SARS-CoV-2 [55,56].

#### 4.1.3. Virus-like Particles (VLPs) Vaccines

**Figure 4:**
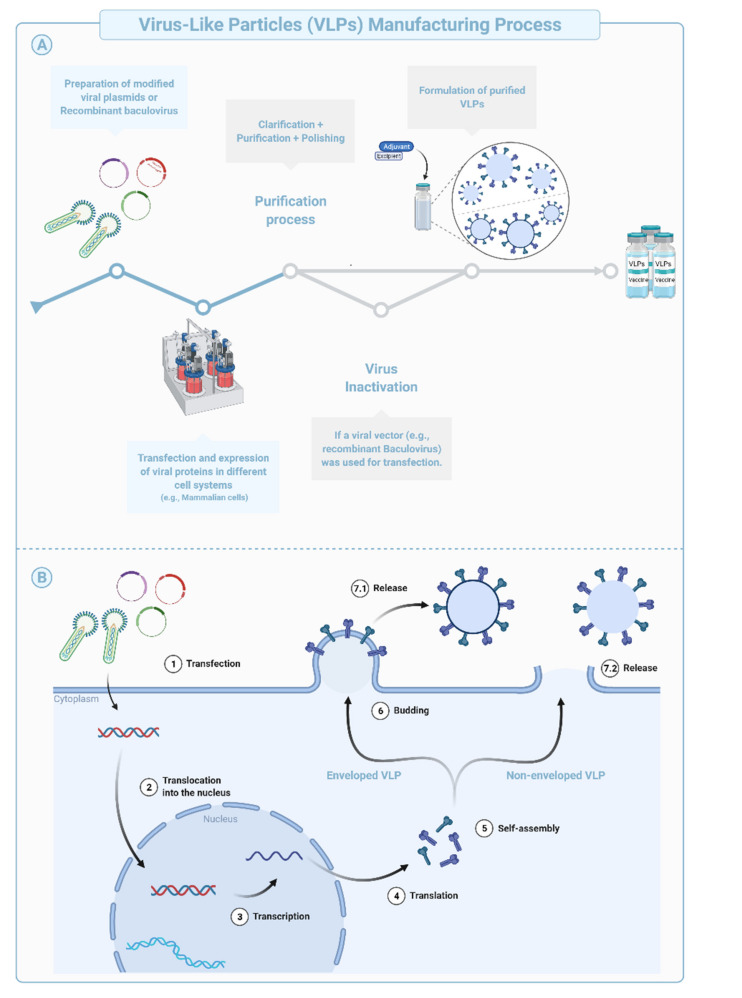
Schematic representation of the production and purification process during manufacturing of Virus-like Particles (VLPs), shows (**A**) the manufacturing process of VLPs and (**B**) their expression in cell systems.

**Figure 5:**
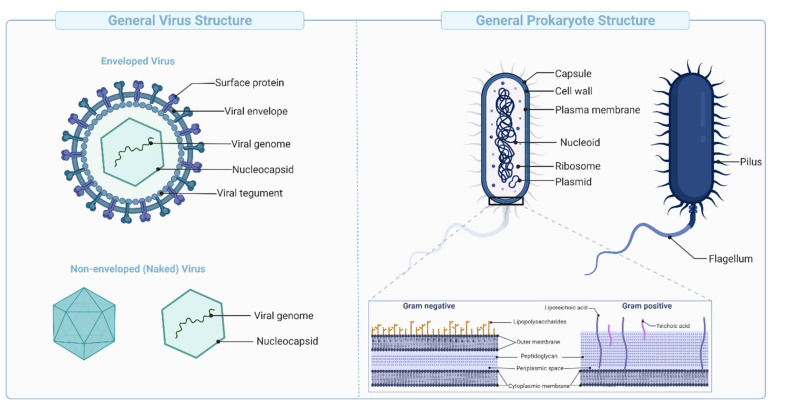
Schematic representation of viral and bacterial structures, showing the typical components of enveloped and non-enveloped viruses (**Left**), and bacteria (**Right**).

Virus-like Particles (VLPs) are macromolecular assemblies designed to mimic the morphology of a native virus (e.g., size, shape, and surface epitopes). VLPs can be subdivided based on the presence or absence of a lipid envelope and the number of protein layers forming the rigid structure known as capsid [57] (Figure 4B, Figure 5). VLP-derived vaccines are typically manufactured in bioreactors following transfection of insect, yeast, bacterial, plant, or mammalian cells with one or multiple genetic constructs (Figure 4A). The constructs encode at least two structural components of the original virus allowing self-assembly into replication-incompetent particles [58,59,60,61]. The immunogenicity of VLPs can be fine-tuned during the design and manufacturing phases using chemical modifications of the surface, addition of immunogenic/dominant peptides and/or adjuvants, or through the choice of the VLP system [62,63]. Precise and targeted modifications of the surface using simple chemistries improve potency, modulate tropism, and allow repurposing of the technology for targeted drug or nucleic acid delivery, imaging (e.g., Positron Emission Tomography and Magnetic Resonance Imaging), and chemical catalysis, among other applications [63].

VLP-based vaccines have been designed to target B cells and induce potent antibody responses following antigen presentation on MHC-II and activation of CD4^+^ cells. VLPs display multivalent epitopes with specific geometries on their surface that facilitate interaction with and crosslinking of B cell receptors (BCR). High-avidity binding with multivalent components of the innate immune system also mediates effective opsonization and uptake by APCs. In vivo studies have shown that the VLPs are actively internalized by different subsets of dendritic cells (e.g., cDC1, cDC2, and follicular DCs), sub-capsular macrophages, and B cells. The macromolecular structure, particulate nature, and nanometer size (e.g., 20–200 nm) facilitate extravasation and rapid draining into the lymphatic system and enable efficient cross-presentation of VLP-derived peptides on MHC-I molecules and subsequent activation of CD8^+^ T cells [64,65].

This technology has been used to develop several licensed vaccines such as the human papillomavirus vaccine (Table 4) and is being explored against Chikungunya, ZIKV, and SARS-CoV-2 viruses [66,67,68]. Compared with other traditional vaccines, the increased potency of this vaccine technology has been attributed to multivalent interaction (increased avidity) with cells of the innate immune system and their subsequent activation. The presence of carry-over agonists (e.g., nucleic acids, and lipids, etc.) naturally packaged during assembly of VLPs also increases the immunogenic potential of these vaccines [59,69]. However, manufacturing challenges in design, purification, and storage impede the practical utility of this technology and increase cost [70].

#### 4.1.4. Synthetic Peptide Vaccines

Immune responses to pathogens are dominated by effector cells that recognize either one or multiple epitopes on an antigen. Identification and synthesis of these immunodominant peptide sequences are used to develop novel vaccine modalities. The design of synthetic peptides involves extensive in vitro screening and modeling (i.e., atomistic interactions) to identify appropriate immunodominant peptides with suitable manufacturing characteristics [71]. Peptide vaccines are synthesized using fragment condensation techniques or solid-phase synthesis, and are subject to stringent purification and characterization [72]. Due to their small size, peptide vaccines are typically mixed with or conjugated to an adjuvant to enhance their immune response and uptake by APCs. Adjuvants must be carefully chosen, since engineered epitopes are sensitive to denaturation or emulsification that might occur in the presence of specific adjuvants.

Peptide vaccines are safer than live-attenuated or killed vaccines [73] and have demonstrated efficacy against infectious [74] and non-infectious diseases (e.g., Alzheimer, and cancer) (Table 5). The control over peptide engineering, synthesis, and quality offers many advantages including comprehensive knowledge of the molecular composition of the vaccine antigens and the ability to elicit a focused, epitope-specific immune response. In addition, rapid modification of sequences to generate strain-specific responses, and the absence of pathogenic/toxic contaminants, are easily achieved using this technology. Synthetic peptides can be modified and/or conjugated to macromolecular structures to reduce unwanted side effects [71] or to improve physicochemical stability and immunogenicity.

However, this vaccine approach presents practical and theoretical difficulties. Restricting the immune response to a few epitopes reduces breadth and selects for effector clones that are unable to respond to escape variants. The use of multiple, or promiscuous T cell epitopes, and the inclusion of B cell epitopes helps cover MHC variation in the population, address antigenic diversity, and increase immune breadth [21,71,75,76]. In addition, since the choice of the epitope is restricted to linear epitopes, conformational B cell epitopes are difficult to mimic. Fortunately, the assembly of peptides on suitable backbones, either to reconstitute the native epitope conformation or to create peptide nanoparticles, has enabled improved immune responses. Antibodies elicited in response to peptides may cross-react with normal tissues, especially if the targeted pathogens display host mimicry to evade the immune response. Despite these practical and theoretical considerations, the synthetic peptide vaccine technology is highly flexible and holds great potential as a standalone strategy or in combination with other technologies.

#### 4.1.5. Fractional Inactivated Vaccines: Toxoid Vaccines

Toxoid vaccines are derived from the inactivation of toxins—a harmful substance produced and secreted by bacteria (not viruses). These vaccines generate an immune response against the disease-causing agent rather than the pathogen itself. Toxins cause several diseases such as diphtheria, tetanus, botulism, cholera, pseudomembranous colitis, etc.

Inactivation is typically mediated by chemical treatments (e.g., formaldehyde) to alter specific amino acids and induce minor conformational changes in the toxin structure. In general, mild inactivation procedures are applied to ablate the biological effects of these toxins while preserving physicochemical properties, overall structure, and immunogenicity [77,78]. Formaldehyde inactivation under specific conditions is superior to heat treatment as it preserves secondary/tertiary structures, improves thermal stability, and reduces aggregation [51]. Physical methods with heat and pH effectively inactivate toxins but tend to decrease immunogenicity and increase aggregation [51,79]. Repeated dosing, and/or formulation with adjuvants such as aluminum salts, may be used to improve the immunogenicity of this vaccine technology.

Immunization with inactivated toxins generates antibody-mediated immune responses that prevent and neutralize cytopathologic effects of bacterial toxins on tissues, reduce bacterial invasiveness, and render the invading microorganism harmless [80]. Since anti-toxin responses typically do not target the bacterium, decolonization (or elimination of the disease-causing bacteria) occurs. The latter is mediated by one or all of the following—engagement of innate immune cells, use of treatment modalities (e.g., antibiotics), and competition between the bacterial pathogen and the normal microbiota. Toxoid-specific T cell responses are mostly restricted to CD4^+^ cells [81] and play an important role in promoting potent antigen-specific B cell responses (including memory B cell response).

Vaccines prepared using the aforementioned methods are safe, stable, and suitable for long-term storage, but need to be formulated with adjuvants in most cases. Toxoid vaccines often induce local injection site reactions that resolves 48–72 h after immunization. These mild side effects are caused by either the adjuvant or type III (Arthus) reaction (Type III reactions results from excess antibody complexing with the injected toxoids and activating the classical complement pathway causing an acute local inflammatory reaction). In conclusion, the optimization of toxin inactivation procedure and choice of adjuvants are key factors in ensuring the success of this technology. Diphtheria and Tetanus Toxoids and Acellular Pertussis (DTaP) vaccines such as Daptacel^®^, Infanrix^®^, and Kinrix^®^, are examples of clinically used toxoid vaccines (Table 3).

#### 4.1.6. Polysaccharide, and Polysaccharide Conjugate Vaccines

These vaccines are derived from carbohydrate-based polymers such as teichoic-acids, peptidoglycans, and glycoproteins, that form the capsular structure of certain bacterial pathogens (Figure 5). Polysaccharide-encapsulated bacteria such as *Haemophilus influenzae*, *Neisseria meningitidis*, and *Streptococcus pneumoniae* cause life-threatening infections such as meningitis, sepsis, and pneumoniae [82]. Several vaccines have been licensed against bacterial capsular polysaccharides, such as Menomune^®^, to provide protection against invasive meningococcal disease (Table 6).

Polysaccharides are potential targets for vaccine development when inactivation methods are ineffective. Since polysaccharides are not processed and displayed on MHC molecules like proteins, immune responses are T cell independent and not mediated by CD4^+^ and CD8^+^ T cells (Section 3). Instead, a particular subtype of B cells in the spleen known as the marginal zone CD21^+^ B cells (MZB) play an essential role in the detection and binding of naked or complement-coated polysaccharide antigens [83]. The interaction between the polysaccharide antigen and the BCR activates B cells to secrete IgM. As discussed in Section 3, T_h_ cells play an important role in the adaptive immune response, and their absence prevents class switching, production of high-affinity IgG, and formation of durable B cell memory responses [84,85].

Despite relative efficacy in adults, this vaccine technology fails to induce protective responses in individuals that are most vulnerable to bacterial infections (e.g., <2 years of age). Infant MZB cells are not fully developed and are therefore unable to recognize bacterial polysaccharides and induce IgM responses [86]. The addition of adjuvants and the formation of glycoconjugates have been used to induce T cell response and improve the immunogenicity of this platform. Polysaccharide conjugates are produced by covalent attachment of the polysaccharide with a carrier protein such as diphtheria or tetanus toxoids (among others) to increase immunogenicity and improve protection in infants and children [87,88]. The mechanism of action of the conjugate vaccine is similar to that of a polysaccharide vaccine. However, in this case, both conjugate protein and the polysaccharide are presented on MHC-II, leading to recognition by TCR and activation of the T_h_ response [77,85]. The interaction between T_h_ and B cells improves titers and the quality of antibodies as well as B cell memory. The detailed mechanism of action of this vaccine technology has been reviewed elsewhere [89]. Examples of FDA-approved polysaccharide conjugate vaccines are listed in Table 7.

##### Next-Generation Vaccine Platforms

The development of vaccines against emerging pathogens with pandemic potential is not feasible using conventional technologies, due to inherent manufacturing issues discussed in the previous section (Conventional (Classical) vaccine technologies). As a result, it is important to develop platforms that can be applied to respond rapidly to biowarfare and pandemic threats and adapted effectively to address emerging escape variants. In addition, such platforms would implement plug-and-play modular design (or recipe-like) to enable rapid response and large-scale manufacturing associated with a smaller footprint, lower cost, and easier deployment in most geographical areas. The development of viral vectors for gene therapy, and the use of nucleic acids to encode antigens (such as mRNA vaccines), coupled with advances in molecular biology, bioinformatics, and technologies such as NexGen sequencing, have enabled the development of novel vaccine platforms that meet the above criteria. However, next-generation platforms are not feasible to produce vaccines using non-protein antigens (such as polysaccharides), since they cannot be encoded by the nucleic acids. Conventional technologies will remain the method of choice to produce such vaccines.

In the following sections, we introduce next-generation vaccine platforms and discuss their mechanisms of action, advantages, and limitations along with examples of approved vaccines.

#### 4.1.7. Bacterial Vectored Vaccines

Using live bacterial cells as carriers has emerged as an intriguing approach to produce novel vaccines [90] with promising results in ongoing research. Bacterial carriers are classified into non-pathogenic and attenuated pathogenic bacteria. Bacteria use the mucous membranes to gain entry for infection, making them suitable for mucosal administration and induction of mucosal immunity. The biggest downside, however, is the risk of infection, especially in children, the elderly, and immunocompromised individuals. As a result, non-pathogenic bacteria such as *Lactobacillus* sp. may be better suited as vaccine vectors [91]. Genetic engineering techniques have enabled the identification and deletion of critical bacterial virulence genes, allowing attenuation of dangerous bacteria such as *Yersinia pestis* to be used as vectors that cannot regain virulence [92].

#### 4.1.8. Viral Vector-Based Vaccines

**Figure 6:**
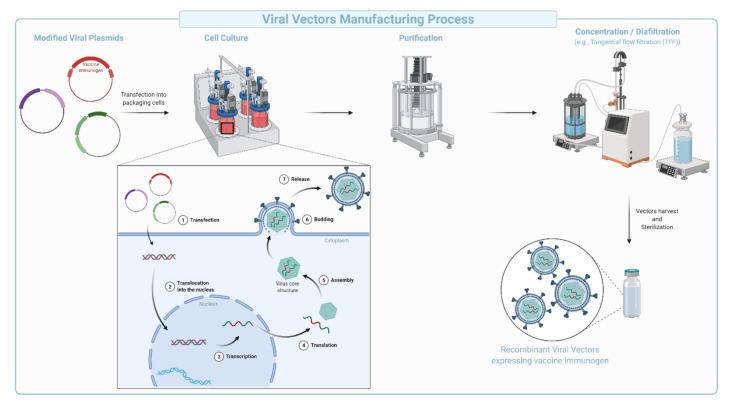
Schematic representation of the production and purification process during manufacturing of viral vectors. Modified viral plasmids that code for the vector components and the vaccine immunogen (transgene) are designed to co-transfect packaging cells. Within the cells, the plasmids are expressed, resulting in viral particles containing the vaccine immunogen. Particles assemble in the cytoplasm and are released into the media via cellular lysis before further purification, concentration, diafiltration, and characterization.

Viral vector-based vaccines are derived from viruses engineered to encode genes for one or several antigens cloned into the vector backbone (Figure 6). Viral vectors can be engineered to be replication deficient (replication incompetent), while maintaining the ability to infect cells and express the encoded antigen. Replication-competent vectors are considered true infections akin to live-attenuated vaccines. The manufacturing of viral vectors has been streamlined in a multistep process comprising of plug-and-play types of genetic engineering approaches, large-scale transfection followed by expansion of cultured mammalian cells, collection, purification, concentration, diafiltration, and formulation (Figure 6). The vesicular stomatitis virus (VSV)-derived Ebola vaccine encoding the Ebola surface glycoprotein (Gp) (Table 8) is an example of a replication competent vaccine. The vaccine was FDA-approved in December 2019 and used in the Kivu Ebola epidemic as part of a ring vaccination strategy. In contrast, replication-incompetent viral vectors do not generate productive infection and are generally safer and easier to manufacture [93]. Replication deficient human and chimpanzee adenoviruses (Ad, and ChAd), Adeno-associated virus (AAV), modified vesicular stomatitis virus, modified vaccinia virus Ankara (MVA), poxvirus, and Newcastle disease virus (NDV), are additional examples of viruses that are being heavily used in the development of safe, viral-based vaccines [94,95].

Typically, this platform mimics natural infection to generate potent humoral and cellular (CD4^+^ and CD8^+^) responses [96]. Strong immune responses observed with this platform are due to broad tropism, the high transduction efficiency of the vectors into target cells, potent antigen expression due to the use of strong promoters to drive transcription, the longevity of antigen expression, and the inherent immunogenicity of the virus used as vector (e.g., presence of pathogen-associated molecular patterns on the vector, or as carry over during production) [94].

Viral vectors are being increasingly used in the production of prophylactic vaccines due to the versatility of the manufacturing platform and the ability for rapid deployment in the event of an epidemic or pandemic. In addition to being highly immunogenic, viral vector-based vaccines are easier to manufacture, and in some instances safer in comparison with the inactivated, live-attenuated, and recombinant protein technologies. Since viral vector-based vaccines induce a strong immune response, they are typically meant for either a single administration or as a component of a mix and match heterologous vaccine regimen [97].

Despite high immunogenicity in preclinical studies, data from clinical trials indicate lower than expected efficacy of the platform. Noticeably, the use of adjuvants positively modulates both immunogenicity and efficacy of tested vaccines [96]. Major caveats of this platform include pre-existing immunity to the viral vector and reduced efficacy of subsequent administrations due to anti-vector immunity. Strategies developed to circumvent such drawbacks include the use of chimeric vectors, vectors from other species (e.g., chimpanzees, cattle, and pigs), or vector serotypes that are known to have low seroprevalence among the human population. Seroprevalence can differ across different regions and needs to be carefully considered during the development of such vaccines. SARS-CoV-2 vaccines were developed using vectors with low seroprevalence such as human adenovirus serotype 26 (Ad.26) used by Janssen/Johnson & Johnson, and chimpanzee adenovirus (ChAd) vector used by Oxford/AstraZeneca (Table 8). The vaccines were well tolerated and demonstrated an overall efficacy of 66% and 75% respectively in preventing symptomatic COVID-19 disease [98,99,100,101]. However, several countries paused vaccination campaigns around March-April 2021 following rare cases of thrombosis with thrombocytopenia syndrome (TTS) affecting some populations [102]. Upon further consideration, major regulatory agencies including the Center for Disease Control (CDC), the US FDA, EMA, and WHO concluded that the benefits of vaccination outweighed the risk significantly due to a very low risk of developing TTS in response to these vaccines [103]. However, these rare clotting events remain a significant concern that needs to be addressed to fully exploit the potential of this platform. The reader is invited to consult this excellent review for more details about the different adenovirus vectors used for the development of SARS-CoV-2 vaccines and a discussion on TTS [104].

#### 4.1.9. Synthetic DNA Vaccines

Since their emergence in the early 1990s [105,106,107,108], the application of synthetic DNA (***syn***DNA) vaccines has been investigated against several pathogens (e.g., HIV, Ebola, HPV, Zika) and is currently being tested in various clinical trials, including SARS-CoV-2 [109,110]. DNA vaccines are large, polyanionic, sensitive to nucleases, and exhibit less efficient passive entry into cells. Delivery methods such as gene gun, jet, electroporation (EP), and nanoparticle-based systems have increased ***syn***DNA uptake in vivo [111]. EP uses short-lived electrical impulses to create transient and reversible permeabilization of cell membranes and increase the nucleic acid uptake by 100–1000× [109,112]. EP also facilitates leukocyte extravasation due to enhanced blood vessel permeability [113]. Portable EP devices are being used in most clinical trials [112].

***Syn***DNA delivered into the muscle is believed to transfect myocytes [114], keratinocytes [115], and tissue-resident APCs. Internalized DNA is translocated into the nucleus, transcribed into messenger RNA (mRNA), and exported for protein translation. The antigen generated can be presented on both MHC-I and II, partially explaining robust T cell response. Tissue-resident APCs expressing the antigen of interest can directly traffic to the draining lymph node to initiate immune responses. On the other hand, antigen expression on myocytes may generate immune responses by translation and secretion (or shedding) of the antigen into the local environment. This promotes the uptake and cross-presentation (MHC-II) by un-transfected APCs. B cells may also recognize secreted/shed protein, leading to their T cell independent activation. Irrespective of being secreted or shed, the soluble antigen can drain to lymph nodes, extending antigen presentation locally and in distal tissues, resulting in improved GC reactions and re-expansion of LN primed CD4^+^ and CD8^+^ T cells. Transfected myocytes upregulate MHC-I and other co-stimulatory molecules such as CD80, and may contribute to T cell responses by priming naïve CD8^+^ T cells [116]. ***Syn***DNA vaccines can induce both humoral and the cellular components of the immune responses with several preclinical and clinical studies demonstrating potent antigen-specific CD4^+^ and CD8^+^ T cell responses [109,117]. The ability to induce both immune responses differentiate this platform from the more conventional technologies described in previous sections of this manuscript (e.g., inactivated virus).

**Figure 7:**
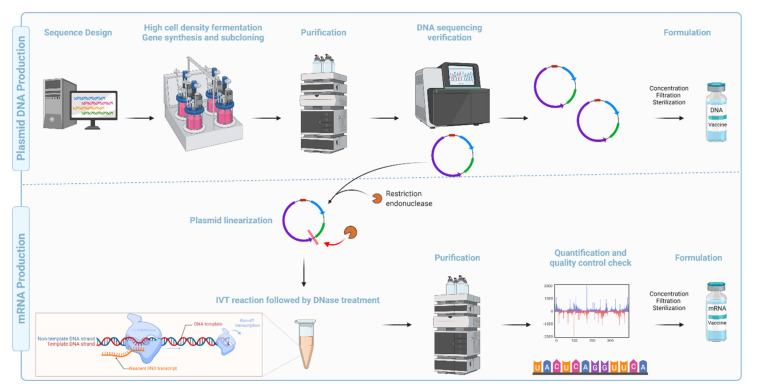
Schematic representation of the production and purification process during manufacturing of DNA and mRNA vaccines. (**Top**): Plasmid DNA production: Designing the sequence is the first step in developing a genomic vaccine followed by high cell-density fermentation, gene synthesis, and subcloning. Cells are harvested, lysed, and purified using chromatography. DNA plasmids are then sequenced for quality assurance before being concentrated, filtered, and sterilized for DNA vaccine formulations. (**Bottom**): mRNA production: mRNA synthesis for RNA-based vaccines requires the linearization of the DNA plasmid to ensure a run-off transcription. Synthesis of mRNA from the DNA plasmid template is catalyzed by an in vitro transcription (IVT) enzymatic process. RNA polymerase (ex. T7 Polymerase), nucleotide triphosphates (NTPs) substrates, polymerase cofactor MgCl_2_, a pH buffer containing polyamine, and antioxidants are all components of the IVT procedure. Following QC check, the mRNA is concentrated, filtered, and sterilized.

Compared with the conventional inactivated, attenuated, and recombinant subunit vaccine platforms, ***syn***DNA vaccines are faster, cheaper, and easier to manufacture [117] (Figure 7). They are also amenable to lyophilization, thermostable, and display high pharmaceutical stability (long-term storage) [118]. Recent advancements in codon optimization, molecular and structural biology, immunoinformatics, immunogen design, and technological advancement in purification methods, along with discovery/development of novel adjuvants, and efficient delivery systems, have improved the potency and safety record for this platform [119,120,121].

Long-lived antigen expression following delivery of ***syn***DNA into the muscle or the dermis, mediates potent immune response due to sustained Tfh responses and GC phenotypes [122,123]. However, the persistence of DNA in the nucleus raises safety concerns since, in theory, it could increase the possibility of integration into genomic DNA (gDNA). However, experimental data suggest that integration into gDNA is only speculative as indicated by extremely rare events that are well below the FDA limit for non-persistence (<100 copies of plasmid/mg of host DNA) [124]. Despite positive clinical data, no DNA-based vaccine is licensed for human use, likely because generation of robust B and T cell responses with this platform requires at least a prime, and two-three booster administration. However, several DNA vaccines have been licensed for veterinary applications, e.g., Melanoma in dogs [125] and West Nile virus in horses. Safe and effective application of these vaccines in animals will likely be instrumental in providing proof-of-concept assisting in eventual application for clinical use in humans.

#### 4.1.10. mRNA Based Vaccines

The concept of mRNA-based therapeutics emerged more than three decades ago when Dimitriadis [126], Malone et al. [127], and Wolff et al. [107] provided the first evidence that endogenously produced (extracted from cells) and in vitro transcribed (IVT) mRNA could be delivered to cells and animals for protein expression. Despite encouraging results from subsequent studies [128,129], major limitations such as potent inflammation and reduced in vivo translation due to mRNA short half-life were quickly recognized. Inflammation-mediated inhibition of protein translation, physicochemical instability, increased sensitivity to nucleases, and poor transfection [130] further limited the potential clinical and therapeutic application (e.g., protein replacement) of the platform. Overcoming these shortcomings significantly improved the platform enabling the successful development of vaccines and/or adjuvants (e.g., CureVac RNActive^®^ platform). Martinon et al. and Conry et al. [129] showed that mRNA loaded into liposomes elicited antigen-specific cytotoxic T lymphocytes (T_c_) and humoral responses paving the way for mRNA vaccine development and early human trials [130].

Recent technological advances, including the incorporation of modified nucleosides into in vitro transcribed (IVT) mRNA [131,132,133] and removal of contaminants using purification chromatography pioneered by Kariko and Weissman [134,135,136], were critical for the development of safe and potent mRNA vaccine platform. Further improvements in sequence engineering and codon optimization [137], and innovations in cap moieties and capping strategies [138], in addition to the evolution of potent and relatively safe delivery systems such as lipid nanoparticles [139,140,141,142,143,144,145], have significantly advanced the development and regulatory approval of mRNA-based vaccines. For instance, nucleoside modifications and elimination of double-stranded RNA contaminants generated during IVT have abrogated the intrinsic adjuvant effect of the IVT mRNA, improved tolerability, and increased antigen/protein expression (translation) by several folds [131,146,147]. Novel cap analogs and capping strategies have increased the yield of properly capped mRNA molecules and alleviated recognition by cytoplasmic innate immune sensors (e.g., RIG-I and MDA5) [148], simultaneously improving translation, safety, and cost of goods. Examples of approved mRNA vaccines are listed in Table 9.

mRNA vaccines can be divided into three major categories: (i) conventional mRNA, (ii) self-amplifying mRNA (SAM), and (iii) circular RNA (***circ***RNA). Conventional in vitro transcribed (IVT) mRNAs are relatively simple in their architecture and manufactured at high yield using a cell-free template-directed enzymatic synthesis [130]. Linearized plasmid DNAs are typically used as templates for mRNA synthesis, and contain a promoter sequence, 5′ and 3′ untranslated regions (UTRs), and the gene of interest. The polyadenine tail (PolyA), an important element in mRNA stability and expression can be engineered into the plasmid or enzymatically added after synthesis. The 5′ cap structure is either co-transcriptionally (e.g., CleanCap™) or enzymatically (e.g., Vaccinia Capping system) added to improve mRNA stability and protein expression, and reduce immunogenicity (e.g., intracellular RIG-I sensing) [149,150].

**Figure 8:**
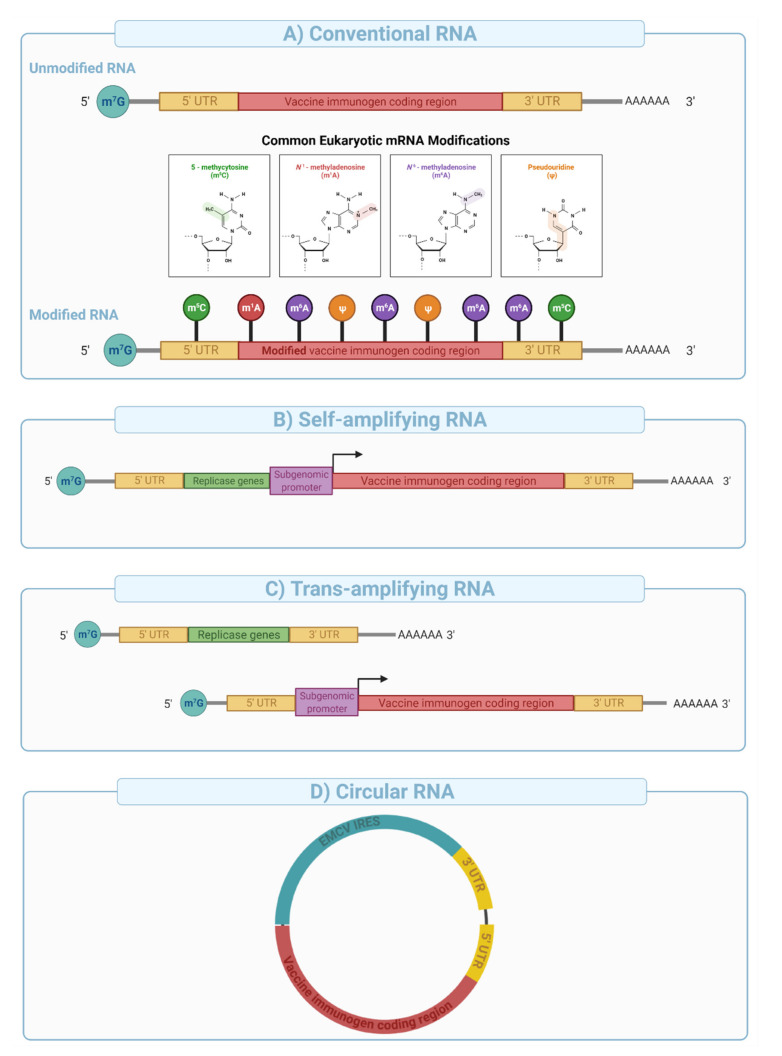
Conventional, self-amplifying, trans-amplifying, and circular RNA vaccine designs. 5′ 7-methylguanosine triphosphate (m7G), 5′ Untranslated region (5′UTR), 3′ untranslated region (3′UTR), and poly A tail are common in all RNA designs. (**A**) Conventional unmodified, and nucleoside modified mRNA encoding vaccine immunogen. (**B**) Self-amplifying RNA encoding replicase gene, a subgenomic promoter, and the vaccine immunogen. Replicase genes (e.g., Alphavirus nsP1-4) code for RNA dependent RNA polymerase complex (RdRP) that recognizes the subgenomic promoter sequences and amplifies vaccine immunogen. (**C**) Trans-amplifying mRNA relies on the same concept of the self-amplifying mRNA but uses two different RNA transcripts: a conventional RNA encoding replicase genes and, an RNA encoding subgenomic promoter along with the vaccine immunogen. (**D**) Circular RNA engineered to enable protein expression through the addition of internal ribosomal entry sites (IRES) (e.g., encephalomyocarditis virus IRES) and/or the incorporation of specific nucleoside modifications in the 5′ UTR.

Depending upon the use of nucleoside modifications during manufacturing and synthesis, the conventional mRNA vaccine platform can be further divided into nucleoside modified or non-modified mRNA (Figure 8A). Nucleoside modifications have proven essential in successful clinical application of conventional mRNA vaccines. The significance of nucleoside modifications in ensuring the success of this platform was indicated by interim data from CureVac that showed disappointing results (47% protection compared to over 94% with the Pfizer/BioNTech and Moderna’s vaccines). This was likely due to the use of unmodified mRNA, which has higher innate immunogenicity than nucleoside-modified mRNA [151], thereby limiting the dose to 12 µg in the CureVac trial compared to 30 and 100 µg for the Pfizer/BioNTech and Moderna trials respectively.

Self-amplifying mRNA is engineered to include viral-derived molecular machines such as alphavirus-derived replicases and conserved sequence elements (CSEs) to enable intracellular amplification of the mRNA sequence [152]. Typical SAM architecture is built from an expression cassette (e.g., sub-genomic promoter and the antigen of interest) cloned between sequences that encode alphavirus-derived nonstructural proteins 1–4 (e.g., VEEV nsP1-4) and a poly adenosine tail (Figure 8B). nsP1-4 proteins assemble into an RNA-dependent RNA polymerase (RdRP) complex that recognizes conserved sequence elements (CSEs) included in the design of the construct (Figure 8B). It then replicates the mRNA vaccine in the cytoplasm, resulting in the efficient and long-lived transcription and protein expression. SAMs are typically large in size (e.g., 6000–12,000 nucleotides), and their manufacturing is more complex and challenging compared with conventional mRNA vaccines due to low yield, difficulty in purification, and susceptibility to autocatalysis and physical degradation.

SAM format is not amenable to nucleoside modification due to impaired interaction between the RdRP and the nucleoside modified sequences resulting in reduced mRNA amplification in target cells [153]. Therefore, potent type I interferon response due to endosomal (e.g., TLR3, 7, and 8) and cytoplasmic sensing (e.g., RIG-1, PKR, etc.) of unmodified nucleosides in SAMs creates a potential hurdle for clinical translation. However, vaccine dosage with SAMs could be 100-fold lower than those used with conventional mRNA vaccines and therefore may offer protection from disease with fewer adverse events in a clinical setting. Preclinical data using a SAM mRNA developed by the Imperial College and Acuitas therapeutic administered at extremely low doses (10 ng, prime boost) showed potent cell and antibody responses in mice [154] and is now under clinical evaluation at doses 300–1000× lower than those used in the approved nucleoside modified mRNA vaccines [141,155].

*Trans-*amplifying mRNAs (*trans*mRNA) prepared by splitting SAM into two different transcripts followed by co-delivery into target cells were introduced for easier manufacturing. In this approach, the nsP1-4 genes are encoded into a separate conventional transcript and co-delivered with a transcript that contains CSEs, subgenomic promoter, and the antigen sequence (Figure 8C). Expression of the nsP1-4 and their subsequent assembly into RdRP allows in-*trans* (on a different molecule) amplification of the antigen encoding transcript. This approach was shown to induce a strong immune response in mice [156] and effectively overcame several limitations of SAMs outlined above.

Circular RNA (*circ*RNA) is a class of non-coding single-stranded RNAs generated through a non-canonical splicing event known as back-splicing in eukaryotic cells [157,158]. ***circ***RNAs have been engineered to enable protein expression through the addition of internal ribosomal entry sites (IRES) and/or the incorporation of specific nucleoside modifications in the 5′ UTR [158] (Figure 8D). This novel platform has been shown to generate potent and stable translation in eukaryotic cells [157] because of extended transcript half-life (e.g., decreased nuclease resistance). Recent studies have suggested that ***circ***RNA can evade intracellular immune sensors such as RIG-I without nucleoside modifications [158]. Qu et al. [159] showed that ***circ***RNA generates potent antigen-specific CD4^+^ and CD8^+^ cellular and humoral immune responses in mice against SARS-CoV-2 and its emerging variants, therefore, providing proof of concept for vaccine applications. 

Immune responses to the mRNA vaccines rely greatly on the delivery system [130], the immunogenicity of the encoded antigen, and the longevity and subcellular localization of antigen expression. Intramuscular and intradermal administration of mRNA vaccines is highly immunogenic and induces local cytokine and chemokine production that initiates prompt recruitment of neutrophils, monocytes, and other cells to prime the immune response/s. Injection of mRNA encapsulated in lipid nanoparticles (mRNA-LNP) has been shown to induce robust infiltration of neutrophils, monocytes, and dendritic cells as well as the activation of pro-inflammatory cytokines (e.g., IL-1β, PTX3, NLRP3, IL-6, GM-CSF) and chemokines (e.g., CXCL-10, CXCL-11, MIP-2) in mice and rhesus macaques [160,161,162]. In contrast to *syn*DNA, mRNA vaccines are directly translated in the cytoplasm, and the ensuing proteins are processed and presented on MHC-I and II, followed by the presentation to CD8^+^ T cells and CD4^+^ T helper cells in the draining lymph nodes (Figure 2). Since mRNA does not need to enter the nucleus, the expression kinetics is much faster, with the onset typically peaking at 4 h after administration.

mRNA vaccines used in preclinical and clinical studies induced T_h1_ skewed responses, and potent induction of antigen-specific germinal center (GCs) and T-follicular helper cells (T_fh_) responses [160,163,164]. In our previous studies, we have shown that the adjuvant activity of the LNP relies on the ionizable lipid component and IL-6 cytokine induction, but not on MyD88- or MAVS-dependent sensing of LNPs [160]. Improved GC reaction and T_fh_ proliferation/activation compared to inactivated and recombinant protein-based vaccines are likely due to the profile and magnitude of the cytokine response induced by the adjuvant (LNP versus traditional adjuvants such as alum or MF59) and a sustained antigen expression up to ten days after intramuscular and intradermal injections [165], leading to longer antigen presentation [123].

In comparison with viral and *syn*DNA vaccine platforms, mRNA presents virtually no risk of integration into the genome. mRNA vaccines are also more cost-effective, and relatively easier to manufacture (Figure 7). Issues with long-term stability at room temperature, dependence on ultra-low cold chain transport, high reactogenicity, and a relatively narrow safety window are major limitations of the platform. The development of potent and biodegradable lipids, as well as new formulations, will most likely address the shortcomings for a new platform.

## 5. Challenges and Opportunities in Vaccine Development

One of the most significant challenges in public health is posed by the emergence of new pathogens with higher transmissibility, fatality rate, or immune evasion potential. Vaccine development against pathogens that evade the immune response (e.g., HIV, Tuberculosis, and malaria) has not been very successful and continues to be an on-going challenge. Genomic variability of certain pathogens, and their ability to rapidly mutate creates challenges for vaccine development, and could lead to evasion, making current vaccines less effective. Depending upon the platform used, next-generation platforms that can be quickly adapted to emerging variants could help resolve this issue to a certain extent. Global surveillance and monitoring efforts are key factors in gaining quick and effective control over pathogens with epidemic and pandemic potential. Partnership efforts such as “Coalition for Epidemic Preparedness Innovations (CEPI)” are instrumental in accelerating the development and equitable access of vaccines. Increased funding and resource provision from central agencies such as National Institute of Allergy and Infectious Diseases (NIAID) are needed to maintain research activities, testing, and development of vaccines. In addition, national, and international efforts to resolve supply chain issues with raw materials, such as those needed for production of mRNA and lipid nanoparticles, along with an increased willingness to share intellectual property and prepared vaccines, will help resolve challenges with vaccine access.

Next-generation platforms could also be used to identify more conserved sequences as immunogens to overcome the antigenic diversity in these pathogens. Moreover, approach of quality by design combining a novel qualitative methodology with a quantitative bioprocess model could be used to enhance robustness and scalability of manufacturing. Finally, the structure and the immunogenic component of a pathogen can be better understood and predicted with the help of machine learning and computational analyses. Artificial intelligence can also help predict the evolution patterns of viruses and help address emerging variants. Generating potent immune response in immunocompromised and older individuals is another field of opportunity for future vaccine development. Better understanding of immune biology, including immunosenescence, inflamm-aging, and selection of adjuvants could drive the development of vaccines capable of generating more potent response in the aged and immunocompromised population. The choice among established adjuvants (i.e., Alum, MF59, CpG, etc.) is relatively limited. Therefore, developing novel and effective adjuvants is becoming increasingly necessary. The immunogenicity can be further improved through modification of the delivery system, engineering of the antigen for higher immunogenicity (i.e., design of nanoparticle forming immunogens or fusion with immunogenic domains, etc.), and modulation of expression.

## 6. Conclusions

Recent widespread emergence of highly infectious diseases such as Ebola, MERS, and SARS-CoV-2 has once again highlighted the importance of vaccination against deadly diseases. In this review, we have discussed several vaccine manufacturing technologies and platforms, both conventional as well as next generation.

Conventional manufacturing technologies have defined the past century of vaccine development, effectively protecting against diseases with high disability and fatality rates such as smallpox, polio, measles, etc. The infrastructure and resources needed for these technologies have been well established and the development costs have been amortized. Though well understood and effective, these technologies are limited by slow, empirical, and expensive development in addition to short-lived protection against several pathogens. Technological advances such as genetic engineering and superior cell-culture techniques could help reduce cost, improve production output, augment knowhow, and enhance their ability to respond faster to emerging threats as we transition to the next-generation vaccine manufacturing platforms.

Next-generation platforms such as mRNA and DNA derived vaccines offer an exciting and promising avenue for vaccine development owing to low costs, safety, high potency, and rapid mass deployment. These platforms are especially relevant for complex pathogens with immune evasion potential. Moreover, unlike conventionally derived vaccines, these platforms are also likely to offer successful solutions for non-infectious diseases such as cancer. Prototype pathogen preparedness can significantly improve response time in the event of a pandemic. Undoubtedly, further funding and effective monitoring of new data will help define a new era of vaccinology and vaccinomics to mitigate present and emerging public health threats.

## Figures and Tables

**Table 1:** List of four major sub-families of Pattern Recognition Receptors (PRRs).

PRRs	Role	Ref
Cytoplasmic Retinoic acid-inducible gene I (RIG-I) like receptors (RLRs)	Key components for pathogenic RNA recognition and modulate an antiviral immune response.	[5,6]
Cytosolic nucleotide-binding oligomerization domain (NOD)- Leucine Rich Repeats (LRR)-containing receptors (NLR)	Regulation of inflammasome reaction and cytokine production.	[7,8]
Toll-like receptors (TLRs)	Represent an essential innate immune sensor, can be largely classified into two categories. Cell surface TLRs: mainly recognize microbial membrane components such as proteins, lipoproteins, and lipids; Endosomal TLRs: mainly recognize pathogen-derived nucleic acids.	[9,10]
C-type lectin-like receptors (CLRs)	Expressed on DCs and have a key role in orchestrating the stimulation of signaling pathways that regulate adaptive immune responses.	[11]

**Table 2:** Examples of FDA-approved live attenuated vaccines.

Pathogen	Vaccine Tradename	Route of Administration	Adjuvant	Manufacturer
Adenovirus Type 4 and 7 Vaccine *	-	Oral	-	Barr Labs, Inc. Montvale, NJ, USA
Measles, Mumps, and Rubella Virus Vaccine	M-M-R^®^ II	Subcutaneous	-	Merck & Co., Inc. Kenilworth, NJ, USA
Tuberculosis (Bacillus of Calmette and Guerin (BCG)) Vaccine	BCG Vaccine	Percutaneous	-	Organon Teknika Corp., LLC. Durham, NC, USA
Cholera Vaccine	Vaxchora^®^	Oral	-	Emergent Travel Health, Inc. Gaithersburg, MD, USA
Dengue Tetravalent Vaccine	DENGVAXIA^®^	Subcutaneous	-	Sanofi Pasteur Inc. Lyon, France
Ebola Zaire Vaccine	ERVEBO^®^	Intramuscular	-	Merck & Co., Inc. Kenilworth, NJ, USA
Influenza Vaccine	FluMist^®^	Intranasal	-	Medimmune, LLC. Gaithersburg, MD, USA
Rotavirus Vaccine	Rotarix^®^	Oral	-	GlaxoSmithKline Biologicals, Brentford, UK
Varicella Virus Vaccine	VARIVAX^®^	Subcutaneous	-	Merck & Co., Inc. Kenilworth, NJ, USA
Smallpox (Vaccinia) Vaccine	ACAM2000^®^	Percutaneous	-	Sanofi Pasteur Biologics Co. Cambridge, MA, USA

* Approved for use in military populations 17 through 50 years of age.

**Table 3:** Examples of FDA-approved inactivated vaccines.

Pathogen	Vaccine Tradename	Route of Administration	Adjuvant	Manufacturer
Poliovirus Vaccine	IPOL^®^	Intramuscularly or subcutaneously	-	Sanofi Pasteur, SA. Lyon, France
Japanese Encephalitis Vaccine	IXIARO^®^	Intramuscular	-	Valneva Austria GmbH. Vienna, Austria
Hepatitis A Vaccine	HAVRIX^®^	Intramuscular	-	GlaxoSmithKline Biologicals. Brentford, United Kingdom
Diphtheria and Tetanus Toxoid Vaccine	-	Intramuscular	-	Sanofi Pasteur, Inc. Lyon, France
Diphtheria and Tetanus Toxoids and Acellular Pertussis Vaccine	INFANRIX^®^	Intramuscular	Aluminum Hydroxide	GlaxoSmithKline Biologicals. Brentford, United Kingdom
Diphtheria and Tetanus Toxoids and Acellular Pertussis Adsorbed and Inactivated Poliovirus Vaccine	KINRIX^®^	Intramuscular	Aluminum Hydroxide	GlaxoSmithKline Biologicals. Brentford, United Kingdom
Diphtheria and Tetanus Toxoids and Acellular Pertussis Vaccine Adsorbed	DAPTACEL^®^	Intramuscular	Aluminum phosphate	Sanofi Pasteur, Inc. Lyon, France

**Table 4:** Examples of FDA-approved Virus-like Particle (VLP) vaccines.

Pathogen	Vaccine Tradename	Route of Administration	Adjuvant	Manufacturer
Human Papillomavirus Bivalent (Types 16 and 18) Vaccine	CERVARIX^®^	Intramuscular	AS04	GlaxoSmithKline Biologicals. Brentford, United Kingdom
Human Papillomavirus Quadrivalent (Types 6, 11, 16, 18) Vaccine	GARDASIL^®^	Intramuscular	Amorphous Aluminum Hydroxyphosphate Sulfate (AAHS)	Merck & Co., Inc. Kenilworth, USA
Human Papillomavirus 9-valent Vaccine	GARDASIL^®^ 9	Intramuscular	AAHS	Merck & Co., Inc. Kenilworth, USA

**Table 5:** Examples of FDA-approved synthetic peptide vaccines.

Pathogen	Vaccine Tradename	Route of Administration	Adjuvant	Manufacturer
Meningococcal Group B Vaccine	TRUMENBA^®^	Intramuscular	-	Pfizer Inc, Inc. New York, USA

**Table 6:** Examples of FDA-approved polysaccharide vaccines.

Pathogen	Vaccine Tradename	Route of Administration	Adjuvant	Manufacturer
Meningococcal Polysaccharide Vaccine, Groups A, C, Y, W-135 Combined	Menomune^®^-A/C/Y/W-135	Subcutaneous	-	Sanofi Pasteur Inc. Lyon, France
Typhoid Vi Polysaccharide Vaccine	Typhim Vi^®^	Intramuscular	-	Sanofi Pasteur SA. Lyon, France

**Table 7:** Examples of FDA-approved polysaccharide conjugate vaccines.

Pathogen	Vaccine Tradename	Route of Administration	Adjuvant	Manufacturer
Haemophilus B Vaccine (Meningococcal Protein Conjugate)	Liquid PedvaxHIB^®^	Intramuscular	-	Merck & Co., Inc. Kenilworth, NJ, USA
Haemophilus b Conjugate Vaccine (Tetanus Toxoid Conjugate)	HIBERIX^®^	Intramuscular	-	GlaxoSmithKline Biologicals. Brentford, UK
Pneumococcal 13-valent Conjugate Vaccine (Diphtheria CRM197 Protein)	Prevnar 13^®^	Intramuscular	Aluminum Phosphate	Pfizer Inc, Inc. New York, NY, USA
Pneumococcal 15-valent Conjugate Vaccine	VAXNEUVANCE^®^	Intramuscular	Aluminum Phosphate	Merck & Co., Inc. Kenilworth, NJ, USA
Haemophilus b Conjugate Vaccine (Tetanus Toxoid Conjugate)	ActHIB^®^	Intramuscular	-	Sanofi Pasteur, SA. Lyon, France
Meningococcal (Groups A, C, Y, W) Conjugate Vaccine	MenQuadfi^®^	Intramuscular	-	Sanofi Pasteur, Inc. Lyon, France

**Table 8:** Examples of FDA-approved viral vector vaccines.

Pathogen	Vaccine Tradename **	Route of Administration	Adjuvant	Manufacturer
Ebola Zaire Vaccine	ERVEBO^®^	Intramuscular	-	Merck & Co., Inc. Kenilworth, NJ, USA
SARS-CoV-2 (Janssen COVID-19 Vaccine) *	JNJ-78436735	Intramuscular	-	Janssen Biotech Inc. Horsham, PA, USA

* Vaccine based on Ad.26 and authorized for emergency use in the United States. ** The Oxford/AstraZeneca vaccine (trade name Vaxzevria^®^) was approved for emergency use by the World Health Organization, and in Europe but has not received FDA approval.

**Table 9:** Examples of FDA-approved mRNA vaccines.

Pathogen	Vaccine Tradename	Route of Administration	Adjuvant	Manufacturer
SARS-CoV-2 (COVID-19) Vaccine *	COMIRNATY^®^	Intramuscular	LNP	Pfizer Inc. New York, USA/BioNTech SE.Mainz Germany
SARS-CoV-2 (COVID-19) Vaccine **	SpikeVax^®^	Intramuscular	LNP	ModernaTx, Inc. Massachusetts, USA

* Comirnaty was granted additional approval for the use in 5–16 years old. ** Authorized for emergency use as of publication date_._

## Glossary

**Abbreviation**	**Definition**
AAV	Adeno-associated virus
Ad	Adenovirus
APCs	Antigen-presenting cells
CD	Cluster of differentiation
CDC	Center for Disease Control
CEPI	Coalition for Epidemic Preparedness Innovations
ChAd	Chimpanzee adenovirus
circRNA	Circular RNA
CLRs	C-type lectin-like receptors
COVID-19	Coronavirus disease 2019
CSE	Conserved sequence elements
DC	Dendritic Cells
dLN	Draining lymph node
DNA	Deoxyribonucleic acid
DTaP	Diphtheria and Tetanus Toxoids and Acellular Pertussis
EMA	European Medicine Agency
EP	Electroporation
FDA	United States Food and Drug Administrations
GC	Germinal center
HIV	Human immunodeficiency virus
IFN-γ	Interferon-gamma
Ig	Immunoglobulin
IL	Interleukin
INF-α	interferon-alpha
IRES	Internal ribosomal entry sites
IVT	in vitro transcribed
LLPCs	long-lived PCs
LNP	Lipid nanoparticle
LRR	Leucine Rich Repeats
MAVS	Mitochondrial antiviral-signaling protein
MBCs	Memory B cells
MERS	Middle east respiratory syndrome
MHC	Major histocompatibility complex
mRNA	Messenger RNA
MVA	Modified vaccinia virus Ankara
MyD88	Myeloid differentiation primary response 88
MZB	Marginal Zone B cells
Mϕ	Macrophages
NAbs	Neutralizing antibodies
NDV	Newcastle disease virus
NIAID	National Institute of Allergy and Infectious Diseases
NLR	Cytosolic nucleotide-binding oligomerization domain (NOD)- Leucine Rich Repeats (LRR)-containing receptors
NOD	Nucleotide-binding oligomerization domain
PAMPs	Pathogen-Associated Molecular Patterns
PCs	Plasma cells
PIZV	Purified inactivated Zika virus vaccine
PolyA	Polyadenine
PRRs	Pattern Recognition Receptors
RdRP	RNA-dependent RNA polymerase
RIG-I	Retinoic acid-inducible gene I
RLRs	Retinoic acid-inducible gene I (RIG-I) like receptors
RNA	Ribonucleic acid
SAM	self-amplifying mRNA
SARS-CoV-2	Severe acute respiratory syndrome coronavirus 2
*syn*DNA	Synthetic DNA
T_c_	Cytotoxic T cells
TCR	T cell receptor
T_fh_	Follicular T helper cells
T_h_	T helper cells
TLR	Toll-Like receptors
transmRNA	*Trans-*amplifying mRNAs
TTS	Thrombosis with thrombocytopenia syndrom
UTRs	Untranslated regions
VLPs	Virus-like Particles
WHO	World Health Organization
ZIKV	Zika virus
